# Muscle: a source of progenitor cells for bone fracture healing

**DOI:** 10.1186/1741-7015-9-136

**Published:** 2011-12-22

**Authors:** Yves Henrotin

**Affiliations:** 1 Bone and Cartilage Research Unit, University of Liège, CHU Sart-Tilman, 4000 Liège, Belgium

**Keywords:** bone repair, fracture, pseudarthrosis, progenitors, muscle

## Abstract

Bone repair failure is a major complication of open fracture, leading to non-union of broken bone extremities and movement at the fracture site. This results in a serious disability for patients. The role played by the periosteum and bone marrow progenitors in bone repair is now well documented. In contrast, limited information is available on the role played by myogenic progenitor cells in bone repair. In a recent article published in *BMC Musculoskeletal Disorders*, Liu *et al*. compared the presence of myogenic progenitor (MyoD lineage cells) in closed and open fractures. They showed that myogenic progenitors are present in open, but not closed fractures, suggesting that muscle satellite cells may colonize the fracture site in the absence of intact periosteum. Interestingly, these progenitors sequentially expressed a chondrogenic and, thereafter, an osteoblastic phenotype, suggestive of a functional role in the repair process. This finding opens up new perspectives for the research of orthopedic surgical methods, which could maximize myogenic progenitor access and mobilization to augment bone repair.

Please see related article: http://www.biomedcentral.com/1471-2474/12/288

## Background

There are millions of fractures each year the world over, generated by traumatic injury. According to the force of the impact, fractures are closed with a limited angle of dislocation, or open with a serious displacement of the broken bone extremities inducing severe injuries of the surrounding tissues (muscles, blood vessels, skin, nerves). While immobilization and surgery may facilitate healing, a fracture ultimately heals through physiological processes in 6 to 8 weeks. Bone repair is dependent on the presence of osteocompetent progenitors that are able to differentiate and generate new bone. Progenitors coming from the periosteum and the bone marrow compartment play a pivotal role. However, there are many circumstances that limit the access of these primary osteoprogenitors, for example, damage to the periosteum, debridement to prevent infection and internal fixation. These circumstances occur in high-energy traumatic open fractures, which often leads to 'non-union' (pseudarthrosis or failed fracture) of the broken bone extremities. In the case of a non-union, a fracture does not heal. The prevalence of non-union of closed tibial shaft fracture is 2.5% and increases 5.7-fold for open fractures with gross contamination and extensive soft damage [1]. Other reasons for a non-union fracture may include complicated, multisegmental fractures (severe comminution), open fractures, fractures associated with tumors (pathologic fractures), infection, insufficient fracture immobilization (fixation), inadequate blood supply, poor nutrition and chronic disease state (diabetes, renal failure, metabolic bone disease). These fractures may lead to severe secondary complications and disability, and result in a high socioeconomical impact. Current treatment requires surgery techniques, but a high percentage of failure is associated with this.

### Current treatment for bone fracture

Most non-union fractures require open surgery to realign the fracture fragments into their normal anatomical position and stabilize the fracture by use of metal plates, rods, screws, and/or wires. Another technique involves the insertion of bone graft material into the surgical site, which stimulates fracture healing. Newer approaches are using recombinant bone morphogenic protein and bone marrow aspirates. Bone marrow may be harvested from the individual's iliac crest and injected directly into the fracture site guided by external imaging (fluoroscopy). Some cases, whether treated surgically or with non-invasive techniques, benefit from the use of electrical, electromagnetic, or ultrasonic stimulation to promote fracture healing and bone growth. Electrical stimulation may be administered by a self-contained device surgically implanted internally at the fracture site or by multiple electrodes placed over the skin near the fracture site [2].

### Myogenic progenitor cells in bone formation

Prevention and treatment of the non-union fracture remains an important field of research. One area of research is better use of local progenitors by improving their migration to the fracture site and their differentiation into bone cells. Therefore, research looking for new progenitors is of the highest importance. In this context, Liu *et al*. [3] have investigated the potential of myogenic progenitors (MyoD-lineage cells) in bone repair in the mouse tibia. They compared the contribution of these cells in the repair of closed tibial fracture (where the periosteum was largely intact), to the repair of open, highly traumatic fracture featuring periosteal stripping and local tissue trauma. Using a MyoD-Cre+:Z/AP+ conditional reporter mouse in which all cells of MyoD lineage are labeled with human alkaline phosphatase reporter, they showed that myogenic progenitors are present in open, but not closed fractures, suggesting that muscle satellite cells may contribute to bone repair in the absence of intact periosteum. This study documents for the first time that muscle cells can play a significant role in bone repair, and offers a new and valuable perspective on the capacity of MyoD-lineage cells to contribute to bone formation and fracture repair. This finding gives a rationale to the use of muscle flaps in orthopedic surgery to treat bone non-union, and advances current knowledge on the beneficial effect of muscle proximity to open-fracture healing. This paper also offers new perspectives for the research of orthopedic surgical methods that could maximize MyoD lineage access and mobilization to augment bone repair.

## Conclusions

Liu *et al*. show for the first time that myogenic progenitors of the MyoD lineage contribute to bone repair, giving new perspectives for treatment of fracture non-union through the optimization of myogenic progenitors proliferation, migration and differentiation.

## Competing interests

The author declares that they have no competing interests.

## Pre-publication history

The pre-publication history for this paper can be accessed here:

http://www.biomedcentral.com/1741-7015/9/136/prepub
